# Pediatric Cardiac Service Development in Croatia

**DOI:** 10.3389/fcvm.2022.793166

**Published:** 2022-02-24

**Authors:** Ivan Malčić, Darko Anić

**Affiliations:** ^1^Department of Paediatric Cardiology, University Hospital Centre Zagreb, Zagreb, Croatia; ^2^Department of Cardiac Surgery, University Hospital Centre Zagreb, Zagreb, Croatia

**Keywords:** congenital heart disease, pediatric cardiology and surgery, mortality, cardiovascular outcome assessment, humanitarian pediatric cardiac assistance

## Abstract

This article describes the establishment of a pediatric cardiac service program in a limited resource country. According to national epidemiological studies about 330 children with congenital heart disease (CHD) are born every year in Croatia. As a part of the former Yugoslavia, there was not an organized strategy for the pediatric cardiac service. After Croatian independence in 1991, even during the war, a need for such service led to the first step in the development of organized care for patients with CHD, a humanitarian mission provided by a non-governmental organization (NGO), from the United States. In the ten-year period (1993–2003), 601 children were operated on during this program. After the end of this program, the Croatian team was not able to cover the whole spectrum of pediatric cardiac care independently. About 60% of the children were sent abroad, and only about 40% of the operations were performed in Croatia. Over the time, the surgical team improved and after a specialized congenital heart surgeon joined, the number and quality of the program in the country raised, and the number of referrals to foreign centers gradually declined. In the meantime, a cardiological interventional program also improved. Today majority of standard congenital heart surgery procedures can be performed in Croatia. Last year our congenital heart team operated on 180 patients with low mortality and the interventional team (pediatric and adult) performed 66 procedures. In the article, we present positive shifts achieved during time as well as weaknesses and reasons for problems in establishing a high-quality CHD center.

## Introduction

The imbalance between rich and poor worldwide is particularly pronounced in pediatric cardiac services. In the third-world countries, more than 80% of children cannot receive adequate cardiac care (1, 2). In addition, in the developed world, there has been an extremely dynamic advance in pediatric cardiology in a theoretical, diagnostic, and therapeutic approach. As part of the former Yugoslavia, Croatia did not have an organized strategy for the development of pediatric cardiac care, so only the children with simpler heart defects were operated on by an experienced adult cardiac surgeon (Professor Josip Sokolić), and the rest were treated medically or referred to other centers, mostly in Yugoslavia. After Croatian independence in 1991, even during the war, the problem of children with congenital heart disease (CHD), become visible. Lack of material resources and good technological support was just a part of the problem. There were no experts at all levels, necessary in the CHD treatment, but also there was no real support of medical and political authorities at this moment, due to much more severe problems caused by the war. Nevertheless, due to the need for this medical service, a humanitarian program was launched, thanks to few enthusiasts. This humanitarian project enabled the beginning of the development of modern pediatric cardiac care in Croatia. We present problems we have encountered and achievements we have accomplished.

## Epidemiology of CHDs in Croatia

The first large epidemiological study on children with congenital heart defects in Croatia was performed from 1995 to 2000. It showed that out of 276,565 newborns, 2,204 had some CHDs (prevalence 8 per thousand). During the first month of life, 50% of heart defects were recognized, and by the end of the first year over 95%, which is a sign of good pediatric care. This study was limited by the fact that it is only population-based and has no features of a clinical epidemiological study. Through it, we learned the dynamics of CHD and associated anomalies, or associated CHD syndromes, and thus opened a scientific path to understand this problem in public health (3). A 4-year clinical epidemiological study followed (2003–2006). During this study, data of clinical course, outcome, and treatment were included and compared with the population register. In that period, 205,051 children were born in Croatia and 1,480 CHD (prevalence of 7.2 per thousand) were recognized (4). From these two population epidemiological studies, with a total of 418,616 newborns over a period of 11 years, 3,684 children with CHD were found (incidence 7.6 per thousand) (Table 1).

**Table 1:** Prevalence of congenital heart disease (CHD) in Croatia (1995–2006).

**Time period**	**Live born children**	**Children with CHD**	**Prevalence (per 1,000 live-born)**
1995	45,800	342	7.5
1996	47,792	370	7.7
1997	47,834	371	7.6
1998	46,536	368	7.9
1999	44,818	404	9.0
2000	43,758	348	7.9
2003	39,668	314	7.9
2004	40,307	289	7.2
2005	42,492	314	7.4
2006	41,446	277	6.6
Total	481,616	3,684	7.6

There is very little difference between the first retrospective population study and the prospective population study lasting what corresponds to the results of most studies conducted in European countries in approximately the same period and of similar duration (5).

## Establishment and Progress of the Unit

### Period 1: Humanitarian Support for the Development of Pediatric Cardiac Surgery in Croatia–10-Year Program

The International Children's Heart Foundation (ICHF) was asked to help to provide the necessary surgery for pediatric cardiac patients. The ICHF team came first time to Zagreb in April 1993, with the help of an American citizen of Croatian descent, and started a program. On that occasion, 14 patients were operated on from the waiting list. They could not be referred to foreign centers due to the extreme cost. Only one child died postoperatively for pulmonary complications. The same, now expanded team returned in August 1993 with richer humanitarian equipment, and operated on 26 children. This was a time when there was no epidemiological study on congenital heart defects and a time when patients were not classified according to the degree of complexity. The humanitarian program of the ICFH lasted from April 1993 to January 2004 (10 years and 9 months). The Croatian government has sponsored the last 5.5 years. Before that, the Croatian association “Big Heart to Small Heart,” which was founded in 1994, together with different humanitarian organizations from the Unites States and Canada gave support to this humanitarian program. During those 10 years, the American cardiac surgery team led by doctor William Novick traveed to Croatia 32 times from April 1993 to July 2003, 601 children underwent primary heart surgeries. Overall mortality for this period was 11% with 16% mortality in the humanitarian program of 151 operations and 8% in the Government-sponsored program of 450 operations (6). The 8% mortality rate is very similar to the 6.3% reported from The International Quality Improvement Collaborative (IQIC) database for CHD (7). In addition, Jenkins et al. report an overall mortality rate of 4.0% for large institutions, whereas the 87 smaller institutions had a mortality rate of 9.8% (8, 9). The results from the second part of this program were similar to those in the United States at about the same time. All modern surgical techniques were introduced in this period, such as the hypoplastic left heart syndrome surgery program. Most of the visits lasted 1–2 weeks, but in the meantime children with a need for emergency procedures were referred to developed centers in neighboring countries (Austria and Germany) and some were sent to Memphis, the United States, regardless of the financial difficulties of the Croatian Health Institute. Some doctors from Croatia spent some period in Memphis, Tennessee, the USA for education, at the expense of the ICHF (cardiac surgeon, anesthesiologist, and a pediatrics intensivist).

When we look back on this period after 20 years, we can conclude that program:

1. Started development of contemporary care for children with CHD in Croatia
2. Improved specific and general skills of staff on many levels (staff learned from our guests, and transferred knowledge to our colleagues and other patients)
3. Raised awareness of a sensitive patient group and their needs (there was a big interest in the topic in press, political and medical societies).

Today we know the weaknesses of the program:

1. There was not a gradual rise of complexity, what is now recommended by all groups involved in helping to establish a CHD treatment program. To cite Dr. Novick: “We begin each trip with RACHS-1 category I and II cases and only once we are certain that all organizational, personnel and equipment issues are solved we will move to category III and IV.” 2 At that time, very complex patients were operated on, mostly after too long delay, which understandably gave poorer results in the early period.
2. The selection of important members of the home team was not a success. In some cases, just the opposite, and that was obvious from the beginning. Some of these people did not have a real interest and the majority left that job in the time that followed.

The program was abruptly terminated secondary to a change in the political control of the country without a plan to continue cooperation, or any other development plan. Partially, the reasons were weaknesses of the program listed above, but also some particular interests, political disagreements, even a mentality, which tends to destroy a potentially successful story.

### Assessment of the Degree of Complexity

Already during the humanitarian period, attention began to be paid to the classification of complexity, and since 2002, it has been used regularly. To assess the degree of complexity, we used two models recognized internationally. The ABC model contains 145 diagnoses, and the overall complexity of heart defects relative to the severity of the surgical approach is rated at 1.5–15 points, divided into four subgroups by type of CHD. The RACHS-1 model is used to assess risk in congenital cardiac surgery, classified according to CHD complexity into six categories (8, 10).

From 2002, the results of the children either who have been operated on by domestic surgeons, or who have been sent for surgery abroad are sent to European Association of Cardiothoracic Surgery (EACTS). Patients were classified into operating groups according to the ABC and RACHS-1 model, and early mortality and prolonged length of stay (PLOS) in intensive care unit (ICU) were analyzed (11).

### Period 2: After Termination of ICHF Visits

After the termination of the assistance program, there was a significant reduction in the number of operations in Croatia. Most patients, especially those with high-risk diseases, were referred to foreign institutions. In the first 5-year period, from 2003 to 2007, 63% of children were operated on abroad and 37% in Croatia (336 vs. 200 patients). Mortality of the children operated on in Zagreb was 4.5%. A long ICU stay was noticed in the relatively low-risk group (Table 2).

**Table 2:** Distribution of cases operated in Croatia by complexity level, mortality, and prolonged length of stay (PLOS), expressed in RACHS-1 category classification (2003–2007).

**RACHS-1**	**Operations *N* (%)**	**Mortality *N* (%)**	**PLOS *N* (%)**
1	47 (23.5)	0 (0)	5 (10.6)
2	68 (34)	5 (7.4)	15 (22.1)
3	80 (40)	5 (6.2)	29 (36.2)
4	5 (2.5)	0 (0)	1 (20)
5	0 (0)	0 (0)	0 (0)
6	0 (0)	0 (0)	0 (0)
Total	200 (100)	10 (5)	50 (25)

In the same period, 336 patients were operated on abroad (Linz, Austria and Munich, Germany predominantly) with similar mortality of 4.16%. It is evident from the data, that a considerable number of patients with lower or intermediate-risk were not operated on in Croatia, but sent abroad. The reason for this, in our opinion, is the insecurity of the team, which was left without the support of experts from abroad, and the pressure of parents, which will be discussed later (Table 3).

**Table 3:** Distribution of cases operated abroad by complexity level, mortality, and PLOS, expressed in RACHS category classification (2003–2007).

**RACHS-1**	**Operations *N* (%)**	**Mortality *N* (%)**	**PLOS *N* (%)**
1	6 (1.8)	0 (0)	0 (0)
2	96 (28.6)	1 (1)	20 (20.8)
3	143 (42.6)	5 (3.5)	32 (22.4)
4	68 (20.2)	5 (7.4)	18 (26.5)
5	3 (0.9)	0 (0)	0 (0)
6	20 (6.0)	3 (15.0)	10 (50)
Total	336 (100)	14 (4.16)	80 (23.8)

In this period, there has been a significant improvement in the economic situation in the country, and there was no restriction in bearing the costs for medical treatment abroad by the National Health Insurance Company. It was the easy way.

**Figure 1:**
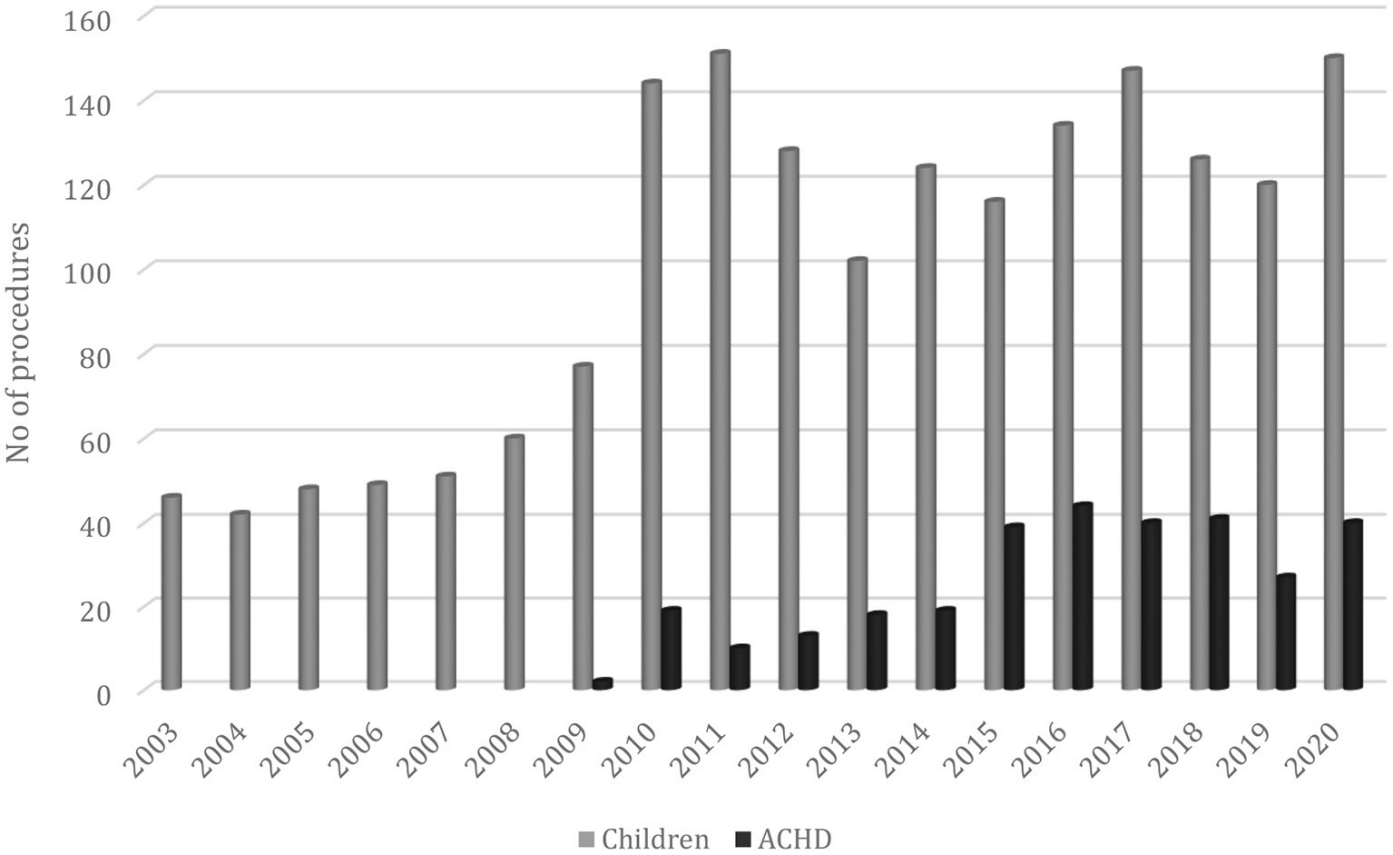
Number of procedures per year in Croatia (2003–2020).

In the following period (2008–2011), the situation improved: 59% of patients were operated on in Croatia and 41% abroad (380 vs. 264 patients) with mortality under 5%. This improvement was partly caused by gaining experience, although development was very slow, due to the small number of operations. Another reason for the upturn was the return of a surgeon, subspecialized in congenital cardiac surgery. With the arrival of a surgeon, the number of operations doubled in the first year (Figure 1).

### Adult Congenital Unit

Survival of pediatric patients with CHD increased over the past decades, and a new group of patients, those who survived CHD into adulthood, emerged. Their need for additional treatment became clear. As a best strategy in the treatment of these patients, specialized centers with multidisciplinary teams were proclaimed. A national adult CHD (ACHD) center was established in Croatia in our institution 6 years ago (12). From that time, the number of operations on patients with ACHD raised, and about 40 patients undergo surgery by the congenital heart team each year, with a low mortality rate (0.96 for the last 10 years). Recently, the number of interventional procedures in these patients also increased. In 2020, 21 percutaneous procedures were performed in patients with ACHD (atrial septal defect (ASD) closure, coarctation stenting, pulmonary valve implantation, etc.). In the future, we expect a further rise of percutaneous treatment, but there will be a place for surgery too, mainly in complex patients (mostly reoperations).

### Current State

In the last 10 years, our CHD team has experienced further progress. Although this has taken much longer than necessary, we are pleased with the current results, but there is still much room for improvement. We now cover all types of conventional congenital heart procedures. As there was already a well-established heart transplant program in our hospital, we launched a pediatric heart transplantation program as well (the first pediatric transplant was done in 2011). Extracorporeal membrane oxygenation (ECMO) is available and used routinely, either due to postoperative stabilization, or as a primary indication for respiratory or cardiac support. We started 3 years ago with Berlin Heart® EXCOR implantation as a longer bridge to the transplantation.

To better illustrate the current situation, we will share with you our results from 2020. Despite the coronavirus disease 2019 (COVID-19) pandemic, there has been no significant deviation from the usual number of procedures in recent years. Last year, 184 patients were operated on and 211 procedures were performed of which 190 were with cardiopulmonary bypass. There were 150 pediatric and 40 patients with ACHD. For comparison, last year 20 patients were sent abroad for surgery, and this is a stable number over the last few years. The majority of these children are referred to the center of their previous operations, some need combined surgical and interventional procedures, several are still considered as a very high risk and the last, some parents prefer an experienced and famous center abroad. We remind you that the Republic of Croatia still covers all the costs of treatment, so parents do not have to pay anything for the treatment abroad. It is unsustainable for an extended time. We hope this number will fall gradually with the rise of our experience. In the same period (2020), pediatric interventional cardiologists performed 45 procedures (Rashkind procedures, congenital aortic valve stenosis valvuloplasty, ASD closure, pulmonary arteries stenting, and percutaneous pulmonary valve implantation) and 18 patients were sent abroad for complex intervention.

Age distribution of the patients operated on in our institution is shown in Table 4. There were no deaths in the operating room. Out of 150 pediatric cases, we have three in-hospital deaths (mortality rate was 2%) (Table 5). In addition, three babies died after only ECMO support (one with Shwachman-Diamond syndrome and myelodysplastic syndrome and monosomy 7, and the other two suffered from cardiomyopathy, on a waiting list for the heart transplant). If we include all the children, the total mortality rate is 3.68%.

**Table 4:** Distribution of the pediatric patients by age.

**Age**	**Patients *N* (%)**
<1 month	25 (16, 67)
1 month−1 year	61 (40, 67)
1–5 years	34 (22, 67)
6–10 years	17 (11, 33)
11–14 years	5 (3, 33)
15–17 years	8 (5, 33)
Total	150 (100)

**Table 5:** Distribution of the pediatric patients operated in 2020 by the complexity level and mortality, expressed in RACHS category classification.

**RACHS-1**	**Operations *N* (%)**	**Mortality *N* (%)**
1	22 (14, 67)	0 (0)
2	63 (42)	0 (0)
3	46 (30, 67)	2 (3, 17)
4	18 (12)	1 (5, 56)
5	0 (0)	0 (0)
6	1 (0, 67)	0 (0)
Total	150 (100)	3 (2)

In the ACHD group, one patient out of 40 died (mortality 2.5%).

Looking at our results and thinking about the current state of congenital heart defects treatment, we consider these are our advantages:

1. There is one institution including all services (National referral center on 4 million inhabitants), which allows for better organization and prevents waste of funds.
2. We now have modern, updated equipment.
3. All costs are covered by the state (National Health Insurance Company although with some struggle, generally provides costs for all medical procedures).
4. Respectable level of staff education and experience has been achieved.

Furthermore, we have problems that still need to be addressed:

1. Shortage of staff (generally there is no interest in demanding professions and jobs, and experts in this field need a very long education and practice).
2. Congenital department is included in the adult cardio-surgical facility (there is competition for spatial and human resources). Pediatric cardiac surgeons cover adult emergency services and the use of the operating room for pediatric patients is limited to 4 days per week. All this limits the total number of operations.
3. Despite the recent effort of pediatric cardiologists, the number of interventional procedures in CHD is still far from optimal. The cause is partly the high cost of devices and partly the shift of generations of pediatric cardiologists.
4. Last but not least, more work is needed to establish good contacts with patients' parents, and if necessary even a public campaign. Despite the good results, parents still insist on sending their children for surgery abroad, which is sometimes difficult to resist. It is a matter of mentality. (If a child dies abroad, that could not be avoided, but when it dies in Croatia, someone must be to blame).

## Conclusion

At the beginning of the 1990's, Croatia was a middle-income country, leading a war for independence with no CHD treatment program. According to epidemiological studies performed in Croatia, 7.2–7.8 children per 1,000 are born with a congenital heart defect yearly, which is similar to other European countries. From 1993, the combination of humanitarian and government-sponsored pediatric cardiac surgical missions was taking place and provided Croatian children with operations for 10 years. In the following period, the care of these patients was left to the home team alone, which was not ready for all these requirements. The care for these children with a large share was provided by established centers in some European countries. From 2008, the team improved, and today it is capable to treat the majority of children and adults with CHD in Croatia. Interventional procedures in this field are also evolving. In this article, we present the development of pediatric cardiac services in Croatia. We are aware that more work needs to be done in the future.

## Data Availability Statement

The original contributions presented in the study are included in the article/supplementary material, further inquiries can be directed to the corresponding author.

## Author Contributions

IM: epidemiological study. DA: discussion. Both authors contributed to the article and approved the submitted version.

## Funding

Funding for this paper is provided by the association Veliko srce malom srcu (eng. Big heart for small heart association).

## Conflict of Interest

The authors declare that the research was conducted in the absence of any commercial or financial relationships that could be construed as a potential conflict of interest.

## Publisher's Note

All claims expressed in this article are solely those of the authors and do not necessarily represent those of their affiliated organizations, or those of the publisher, the editors and the reviewers. Any product that may be evaluated in this article, or claim that may be made by its manufacturer, is not guaranteed or endorsed by the publisher.
